# Development, Application and Utility of a Machine Learning Approach for Melanoma and Non-Melanoma Lesion Classification Using Counting Box Fractal Dimension

**DOI:** 10.3390/diagnostics14111132

**Published:** 2024-05-29

**Authors:** Pablo Romero-Morelos, Elizabeth Herrera-López, Beatriz González-Yebra

**Affiliations:** 1 Department of Research, State University of the Valley of Ecatepec, Ecatepec 55210, México State, Mexico; pablo.r.morelos@gmail.com (P.R.-M.); eliza_herrera83@yahoo.com.mx (E.H.-L.); 2 National Laboratory of Artificial Intelligence and Data Science, CONAHCyT (LNC-IACD), Ecatepec 55210, México State, Mexico; 3 Department of Medicine and Nutrition, Division of Health Sciences, University of Guanajuato, Campus León, León 37670, Guanajuato, Mexico

**Keywords:** melanoma, dermatological lesion, fractal dimension, machine learning, artificial intelligence

## Abstract

The diagnosis and identification of melanoma are not always accurate, even for experienced dermatologists. Histopathology continues to be the gold standard, assessing specific parameters such as the Breslow index. However, it remains invasive and may lack effectiveness. Therefore, leveraging mathematical modeling and informatics has been a pursuit of diagnostic methods favoring early detection. Fractality, a mathematical parameter quantifying complexity and irregularity, has proven useful in melanoma diagnosis. Nonetheless, no studies have implemented this metric to feed artificial intelligence algorithms for the automatic classification of dermatological lesions, including melanoma. Hence, this study aimed to determine the combined utility of fractal dimension and unsupervised low-computational-requirements machine learning models in classifying melanoma and non-melanoma lesions. We analyzed 39,270 dermatological lesions obtained from the International Skin Imaging Collaboration. Box-counting fractal dimensions were calculated for these lesions. Fractal values were used to implement classification methods by unsupervised machine learning based on principal component analysis and iterated K-means (100 iterations). A clear separation was observed, using only fractal dimension values, between benign or malignant lesions (sensibility 72.4% and specificity 50.1%) and melanoma or non-melanoma lesions (sensibility 72.8% and specificity 50%) and subsequently, the classification quality based on the machine learning model was ≈80% for both benign and malignant or melanoma and non-melanoma lesions. However, the grouping of metastatic melanoma versus non-metastatic melanoma was less effective, probably due to the small sample size included in MM lesions. Nevertheless, we could suggest a decision algorithm based on fractal dimension for dermatological lesion discrimination. On the other hand, it was also determined that the fractal dimension is sufficient to generate unsupervised artificial intelligence models that allow for a more efficient classification of dermatological lesions.

## 1. Introduction

As the largest organ in the human body, the skin is susceptible to a wide range of alterations. However, the identification and classification of these lesions can be a challenging task for physicians, especially non-specialists. This difficulty arises primarily from the low contrast between each lesion and the surrounding skin tissue, the visual similarities between different types of lesions and observation artifacts (e.g., bubbles, hair, blood vessels) [1,2].

The accurate classification of skin lesions is a powerful tool for the early detection of different dermatological diseases, including skin cancer. In developed countries, skin cancer is the most prevalent type of cancer according to the World Health Organization. Early detection is crucial as it can lead to a 93% five-year survival rate. However, while various types of skin cancer exist, melanocytic cancers are the most challenging to identify and treat. Although they only represent 4% of skin cancers, they have a mortality rate of up to 80% and a five-year survival rate of only 14% [3].

Melanoma is a neoplastic disease originating from melanocytes, predominantly affecting the skin in 90% of cases (cutaneous malignant melanoma) [4,5]. 

The incidence of melanoma is on the rise, and in Mexico, it ranks third in frequency among skin cancers (14.1%), following basal cell carcinoma and squamous cell carcinoma, according to estimates from the International Agency for Research on Cancer in 2012 [6,7].

Melanomas undergo two growth phases, radial and vertical. During the radial growth phase, malignant cells proliferate radially within the epidermis. Over time, most of the melanomas progress to the vertical growth phase, during which malignant cells invade the dermis and acquire the ability to metastasize. In this context, the depth of lesion invasion can be measured using two main parameters: the Clark levels, providing a qualitative measurement, and the Breslow index, offering a quantitative measurement. The latter index measures depth in millimeters from the granular layer of the epidermis to the deepest point of the tumor lesion across the various layers of the skin [8,9].

The identification and diagnosis of melanoma are attributed to the features encapsulated in the “ABCDE of melanoma”; A—Asymmetry; B—Irregular Border; C—Color Variations; D—Diameter exceeding 6 mm and E—Elevated Surface [10,11]. 

The typical accuracy in diagnosing skin cancers through manual examination of dermoscopic images ranges from 60% to 80%. This accuracy level can differ among dermatologists depending on their level of experience. Research suggests that a dermatologist with three to five years of experience may achieve approximately 60% of accuracy, while those with 10+ years of experience show a 20% improvement in accuracy [12].

In this sense, the identification of melanoma remains challenging even for an experienced dermatologist, with histopathological examination standing as the current gold standard [9,13,14,15]. The challenges in early melanoma diagnosis have spurred the quest for new tools to facilitate this process, including machine learning and mathematical tools, particularly the fractal dimension [16,17,18]. 

Fractal dimension is a mathematical parameter that quantifies the complexity and irregularity of non-Euclidean polygons. Although various studies to date have established the utility of fractal dimension in melanoma diagnosis, its integration with diagnostic algorithms remains an area of active investigation [11,19,20]. 

On the other hand, various models of artificial intelligence, machine learning and deep learning have been developed, which have provided significant evidence to be considered useful for diagnostic processes, especially for dermatological lesions. However, most algorithms based on deep learning and machine learning utilize dermatoscopic images for the development and training of their algorithms based on supervised machine learning [12,21]. 

In this regard, there are some inherent limitations to supervised machine learning models. Firstly, deep learning models require robust computational processes, which unfortunately are not accessible to everyone at present, especially for clinical application rather than research purposes. Additionally, these models require high-quality dermatoscopic images (especially for training convolutional neural networks), which are not easy to obtain, considering that primary care physicians typically do not have such devices [22,23,24]. Thirdly, supervised machine learning models greatly limit the machine’s ability to find underlying characteristics that facilitate the classification of different types of dermatological lesions, confining their capacity to training based solely on characteristics that, as humans, we find relevant, thereby diminishing the great analytical and information-processing capacity of unsupervised machine learning models [25,26,27]. 

Furthermore, it is important to mention that unsupervised machine learning models do not require specialized equipment or large computing capabilities, which facilitates their potential translation into clinical practice, allowing even non-specialized physicians to employ such technologies, even at the primary care level. Therefore, this study proposes the use of fractal dimension as an objective, quantitative and reproducible metric that has proven to be effective in diagnosing melanoma, for its use as a discriminating factor by an unsupervised machine learning model. Thus, the objective of this study was to determine the combined utility of fractal dimension and unsupervised low-computational-requirements machine learning models in classifying melanoma and non-melanoma lesions.

## 2. Materials and Methods

Dermatoscopic records were obtained from the gallery archive of the open-source International Skin Imaging Collaboration (ISIC), and only images captured with a dermatoscope that exhibited no black borders (pixel-free) were selected. The dermatoscopic images were categorized based on their histopathological diagnosis into non-metastatic melanomas, metastatic melanomas, atypical melanocytic proliferation, nevi, verrucous lesions, vascular lesions, squamous cell carcinomas, neurofibroma, basal cell carcinomas, Breslow 1 lesions, Breslow 2 lesions and Breslow 3 lesions. Additionally, lesions were further classified into benign (nevi, warts, neurofibromas and vascular lesions) and malignant lesion (Breslow lesions, melanoma, metastatic melanoma, basal cell carcinoma and atypical melanocytic proliferations) and later in melanomas (Ms) and non-melanomas (NMs). In the same way, only melanoma lesions were subsequently classified into metastatic melanomas (MMs) and non-metastatic melanomas (NMMs). 

**Figure 1:**
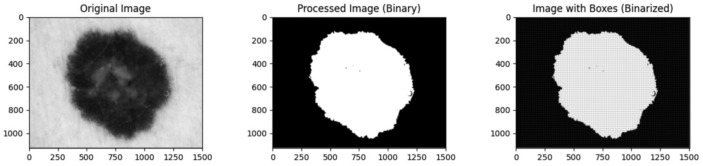
Image-processing pipeline. The image was transformed to 8-bits grayscale and after binary transformation and background elimination were performed and cover it with the maximum number of boxes.

All images included in this study underwent fractal dimension analysis using custom-built Python software v3.10.9, employing several libraries including os, numpy, pandas, PIL (Python Imaging Library), tkinter and tqdm. To achieve this, the images were transformed into 8-bit grayscale bitmaps at a resolution of 512 × 512 pixels. Subsequently, a binary transformation and background elimination were performed (Figure 1). 

Following these preprocessing steps, boxes of varying sizes were drawn within the lesion, and the maximum number of boxes required to cover the lesion and their sizes were calculated for substitution in the following formula:

$$D_{b}=\underset{\epsilon \to 0}{\lim}\frac{logN(\epsilon )}{\log(\frac{1}{N\left(\epsilon \right)})}$$

where *D_b_* represents the fractal dimension of the box counting, *ϵ* is the size of the boxes and *N* is the maximum number of boxes required to completely cover the margin of the analyzed image. 

Fractal dimension values were stored in a Pandas Data Frame and subsequently exported in an Excel file.

To ascertain whether significant differences existed among the fractal dimension values for various histopathologically categorized lesions or based on their Breslow index, Kruskal–Wallis tests with Dunn’s multiple comparison were conducted. Similarly, to identify significant differences between melanoma and non-melanoma or metastatic melanoma and no metastatic melanoma dermatoscopies, a Mann–Whitney U test was employed. 

On another note, ROC curves were generated to determine the sensitivity and specificity of fractal dimension for discriminating between melanoma and metastatic melanoma. All statistical analyses were performed in Graph Pad PRISM v.9.0 for MacOS, Boston, MA, USA, with a *p* < 0.05 significance and confidence intervals of 95%.

Additionally, to use low-computational-requirements machine learning models, principal component analysis (PCA) and k-means analysis were performed. PCA was analyzed in Graph Pad PRISM v.9.0 for MacOS, Boston, MA, USA, from lesions fractal values according to histopathological diagnosis classification, malignant or benign classification, M or NM classification and MM or NMM classification.

K-means clustering models were run using custom-built Python software, employing the libraries tkinter, pandas, seaborn and matplotlib.pyplot. For the analysis, a graphical user interface (GUI) was developed using tkinter to facilitate the selection of an Excel file containing fractal values data. From the dataset, we extracted two key features: the qualitative diagnosis (Dx) and the fractal dimension (FD). To enable numerical analysis, we encoded the qualitative diagnosis using the LabelEncoder from the sklearn.preprocessing module. Next, we applied the K-Means clustering algorithm to the dataset to identify distinct clusters within the data, and all K-means clustering analyses were performed with 100 iterations. 

Encoded qualitative diagnosis was based on histopathological diagnosis classification, malignant or benign classification, M or NM classification and MM or NMM classification.

For evaluating the K-means clustering results, we calculated the inertia and the silhouette scores. These metrics were computed using functions available in the sklearn.metrics module.

To visualize the K-means clustering outcomes, we utilized seaborn and matplotlib.pyplot to generate scatterplots. 

## 3. Results

### 3.1. Dermatoscopic Records

A total of 43,966 dermatoscopic records were obtained from the open-source International Skin Imaging Collaboration (ISIC) from the gallery archive.

Images not captured with a dermatoscope or those containing black borders (pixel-free) were excluded, resulting in the exclusion of a total of 4696 images. This led to a final dataset of 39,270 dermatoscopies.

Based on histopathological diagnosis, the dataset comprised 5858 non-metastatic melanomas, four metastatic melanomas, 99 atypical melanocytic proliferation lesions, 28,778 nevi, six verrucous lesions, 259 vascular lesions, 687 squamous cell carcinomas, seven neurofibromas, 3399 basal cell carcinomas, 150 Breslow 1 lesions, 14 Breslow 2 lesions and nine Breslow 3 lesions. Additionally, the various dermatological lesions were categorized into benign (29,050) and malignant (10,220) lesion and, subsequently an additional classification was performed into melanomas (Ms) and non-melanomas (NMs), resulting in totals of 6035 and 33,235 lesions, respectively. All melanoma lesions were consequently classified as either metastatic melanoma (MM) or non-metastatic melanoma (NMM), with respective counts of 4 and 6031.

### 3.2. Fractal Dimension Analysis in Histopathological Classification of Dermatologic Lesions

**Figure 2:**
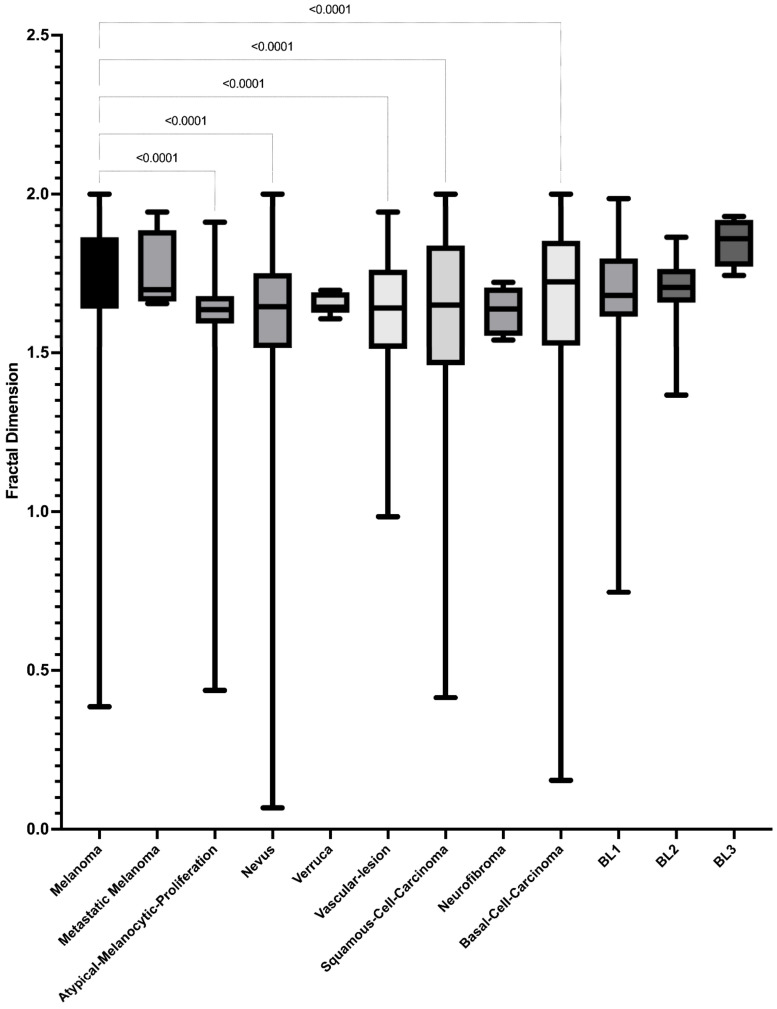
Distribution of fractal dimension values of dermatologic lesion according to histopathological classification.

According to the fractal dimension of lesions classified by histopathological diagnosis, lesions with a higher fractal dimension were associated with Breslow 3 (median 1.859, 95% CI 1.793–1.902), while lesions with the lowest fractal dimension values were linked to atypical melanocytic proliferation (median 1.636, 95% CI 1.582–1.651). However, to determine whether significant differences existed between the fractal dimension values of different lesion types, a Kruskal–Wallis analysis was conducted, yielding a significant result (*p* < 0.0001). Subsequently, a post hoc analysis was performed to identify significant differences between the fractal dimension values of various lesions compared to melanoma lesions. Significant differences were found in squamous cell carcinoma lesions (*p* < 0.0001), vascular lesions (*p* < 0.0001), nevi (*p* < 0.0001) and basal cell carcinoma (*p* < 0.0001) (Figure 2).

#### 3.2.1. Principal Component Analysis for Dermatological Discrimination According Fractal Dimension

Furthermore, a principal component analysis (PCA) was conducted to evaluate the underlying structure of dermatological lesion characteristics, utilizing fractal dimension as a relevant measure. The analysis revealed that lesions such as “BL3”, “Neurofibroma”, “Nevus” and “Melanoma” had significant positive loadings, suggesting similarities in associated characteristics. In contrast, lesions like “Melanoma-metastatic”, “Verruca” and “Basal-Cell-Carcinoma” had significant negative loadings, indicating marked differences from other lesions.

The “BL2” lesion stood out with a significant positive loading, suggesting distinctive associated characteristics. Conversely, lesions of “Atypical-Melanocytic-Proliferation” had a significant negative loading, indicating characteristics opposite to those of “BL2”.

**Figure 3:**
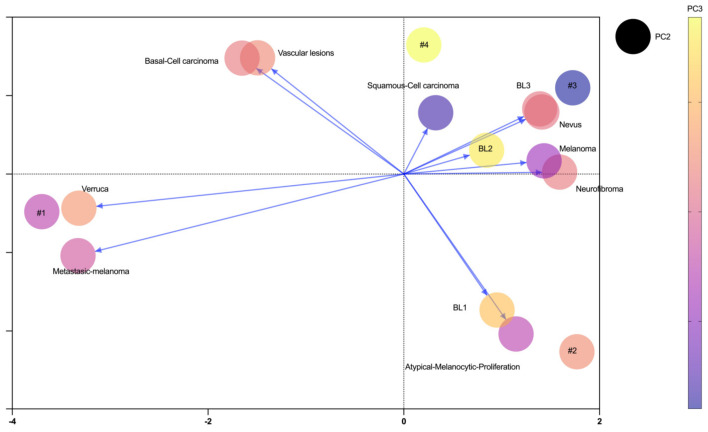
Principal component analysis of dermatologic lesions according to histopathological classification. x axis corresponds to PC1; y axis corresponds to PC2 and color gradient corresponds to PC3. PC = principal component.

The “BL2” lesions exhibit a significant positive loading in PC3, emphasizing specific characteristics. Additionally, lesions of “Squamous-Cell-Carcinoma” show a significant negative loading, suggesting differences in characteristics compared to “BL2” (Figure 3).

#### 3.2.2. K-Means Analysis for Dermatological Discrimination According to Fractal Dimension

**Figure 4:**
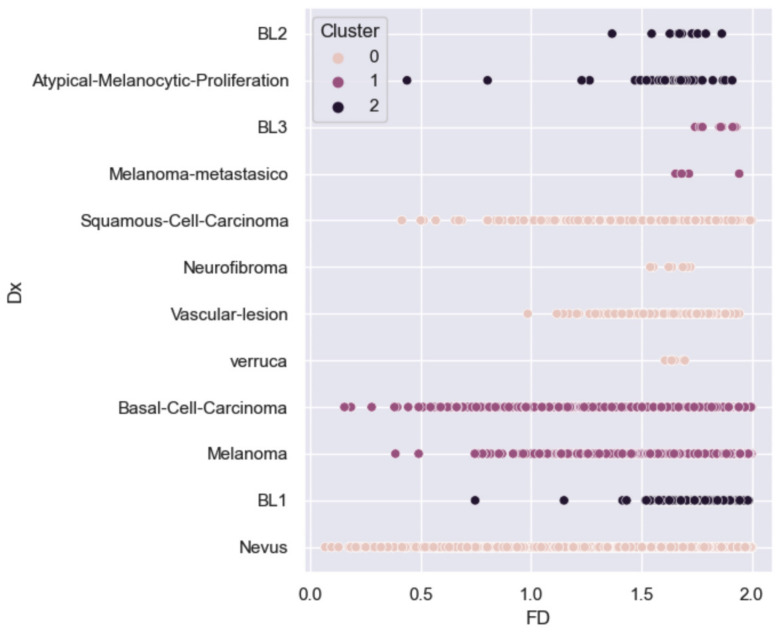
K-means clusterization of dermatological lesion according to histopathological classification. Color keys represent the different centroid aggrupations. FD = Fractal dimension; Dx = Histopathological lesion diagnosis.

Subsequently, to validate the clustering data obtained through the PCA based on fractal dimension, a lesion classification was performed using unsupervised machine learning with the K-means technique, considering three grouping centroids to simulate the PCA-obtained data. Similar characteristics were found among Breslow 1, 2 and atypical melanocytic proliferation lesions. Another group included Breslow 3 lesions, melanoma, metastatic melanoma and basal cell carcinoma. The third group comprised squamous cell carcinoma, neurofibroma, vascular lesions and warts. The groupings were achieved with a strong differentiation between clusters, exhibiting a silhouette index of 0.89 and an inertia of 5848.3 (Figure 4).

### 3.3. Fractal Dimension Analysis for Malignant or Benign Lesion Classification

**Figure 5:**
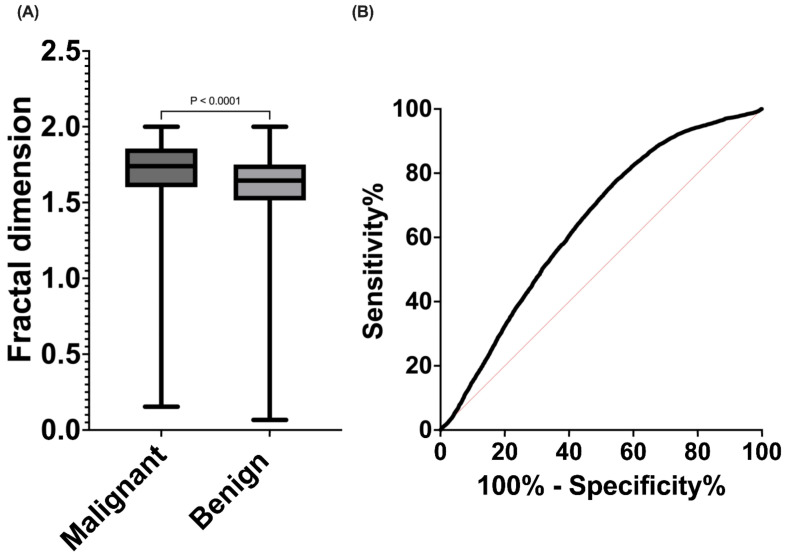
(**A**) Fractal dimension distribution values according benign or malignant lesion classification. (**B**) ROC analysis of fractal dimension for malignant or benign lesion discrimination.

Later, a classification of different types of lesions was conducted, distinguishing between malignant (*n* = 10,220) and benign (*n* = 29,050) lesions. Through a U Mann–Whitney analysis, statistically significant differences were observed (*p* < 0.0001), with malignant lesions displaying higher values of fractal dimension (median 1.74, 95% CI 1.690–1.698) (Figure 5A). A ROC curve was generated, yielding a sensitivity of 72.4% and specificity of 50.1% (Figure 5B).

#### Principal Component Analysis and k-Mean Analysis for Malignant or Benign Lesion Discrimination According to Fractal Dimension

**Figure 6:**
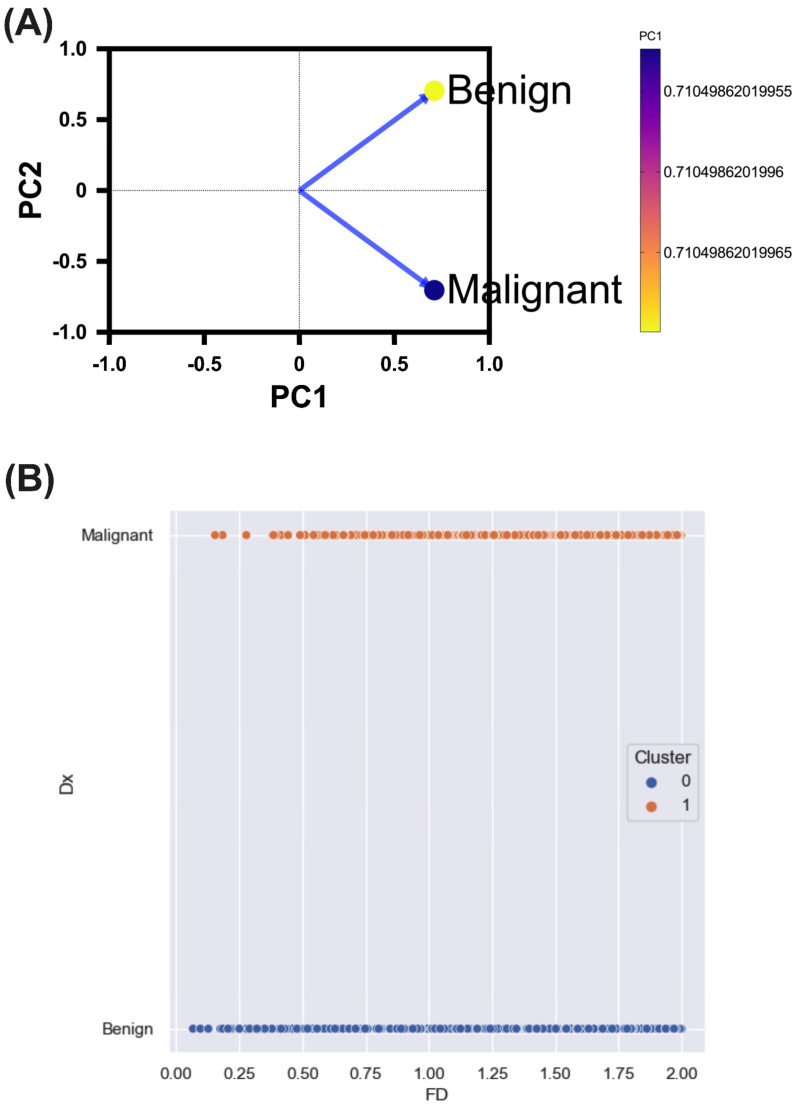
Benign and malignant lesion classification methods. (**A**) Principal component analysis. A clear separation between the two lesions kind groups is observed along the PC2 axis. (**B**) Dot plot showing the distribution of benign and malignant lesions in the different clusters of the k-means analysis. Cluster 0 contains mainly benign lesions, while cluster 1 contains mainly malignant lesions. PC = Principal component; Dx = Lesion classification into malignant or benign lesions; FD = Fractal dimension value.

Subsequent principal component analysis (PCA) revealed two principal components, distinctly segregating between malignant and benign lesions, with loading values of 0.710 (PC1) and −0.704 (PC2) for malignant lesions, and 0.710 (PC1) and 0.704 (PC2) for benign lesions (Figure 6A). To validate these findings, clusterization using k-means was conducted, using two grouping centroids, demonstrating a clear differentiation between malignant and benign lesions through the machine learning approach, with a strong silhouette index (0.79) and inertia of 1851.6, suggesting a high fidelity in discriminating between benign and malignant lesions (Figure 6B).

### 3.4. Fractal Dimension Analysis for Melanoma or Non-Melanoma Lesion Classification

**Figure 7:**
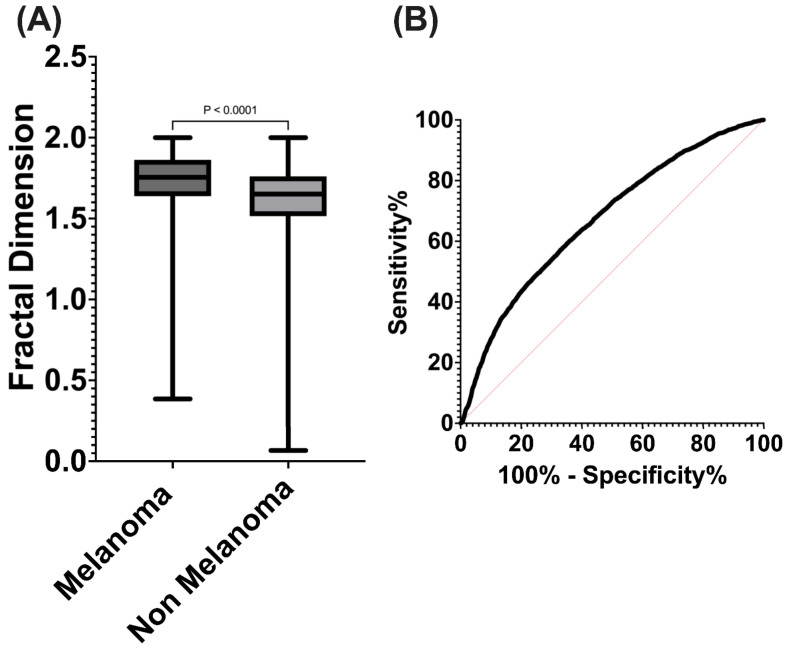
(**A**) Fractal dimension distribution values according to melanoma or non-melanoma lesion classification. (**B**) ROC analysis of fractal dimension for melanoma or non-melanoma lesion discrimination.

Furthermore, for the M (*n* = 6035) and NM (*n* = 33,235) classifications, a Mann–Whitney U test was conducted to determine whether there were significant differences in their fractal dimension. The M group was found to have a significantly higher fractal dimension (median 1.755, 95% CI 1.726–1.734) compared to the NM group (median 1.651, 95% CI 1.611–1.616) with *p* < 0.0001 (Figure 7A). Subsequently, sensitivity and specificity of the fractal dimension were found between M and NM, resulting in an area under the curve of 0.67 (95% CI 0.6627–0.6774, *p* < 0.0001) with a sensitivity of 72.8% and specificity of 50%, using a classification threshold >1.755 for fractal dimension (Figure 7B).

#### Principal Component Analysis and k-Mean Analysis for Melanoma or Non-Melanoma Lesion Discrimination According to Fractal Dimension

**Figure 8:**
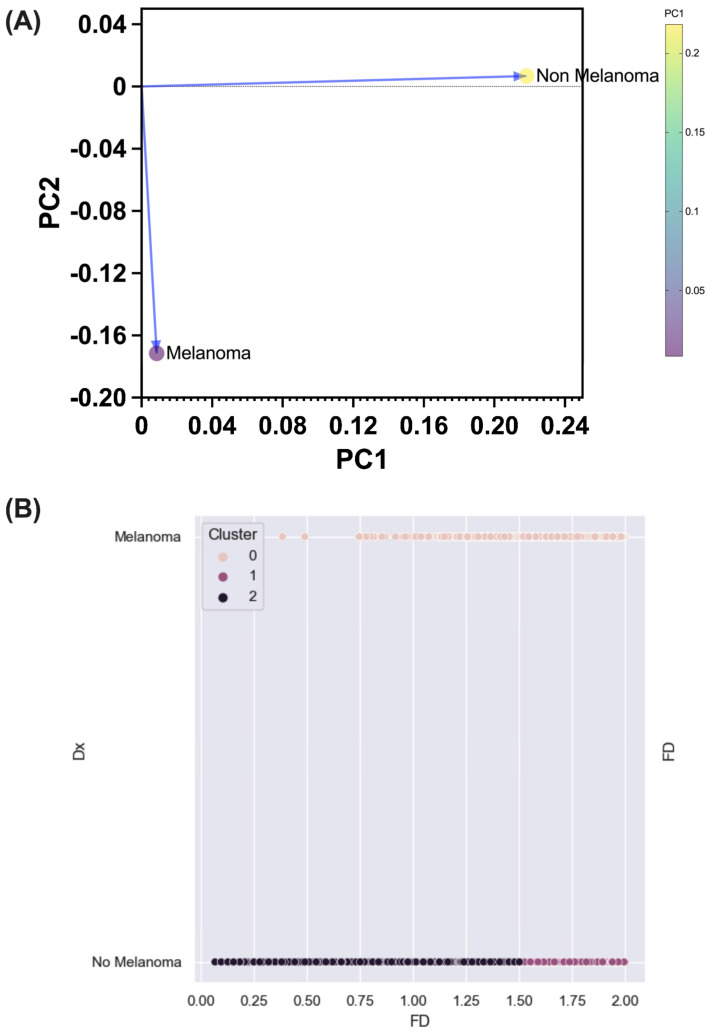
Melanoma and non-melanoma lesion classification methods. (**A**) Principal component analysis. A clear separation between the two lesion groups is observed along both PC axis. (**B**) Dot plot showing the distribution of melanoma and non-melanoma lesions in the different clusters of the k-means analysis. Cluster 0 contains mainly melanoma lesions, while clusters 1 and 2 contain non-melanoma lesions. PC = Principal component; Dx = Lesion classification into melanoma or non-melanoma lesions; FD = Fractal dimension value.

Additionally, PCA between Ms and NMs yielded two principal components. The first component (PC1) had a positive coefficient for both M lesions (0.011) and NM lesions (0.218). The second component (PC2) showed a negative coefficient for M lesions (−0.171) and a positive coefficient for NM lesions (0.008). Therefore, the M and NM categories are primarily separated along the PC1 direction, with NM contributing significantly to the variability in that direction. This demonstrates a differential grouping of each lesion, allowing their separation based solely on the obtained fractal dimension (Figure 8A).

K-means learning process, within two grouping centroids, shows a clear separation of both lesion types with a silhouette index of 0.79 and an inertia of 1836.3 (Figure 8B).

### 3.5. Fractal Dimension Analysis for Metastatic Melanoma or Non-Metastatic Melanoma Lesion Classification

**Figure 9:**
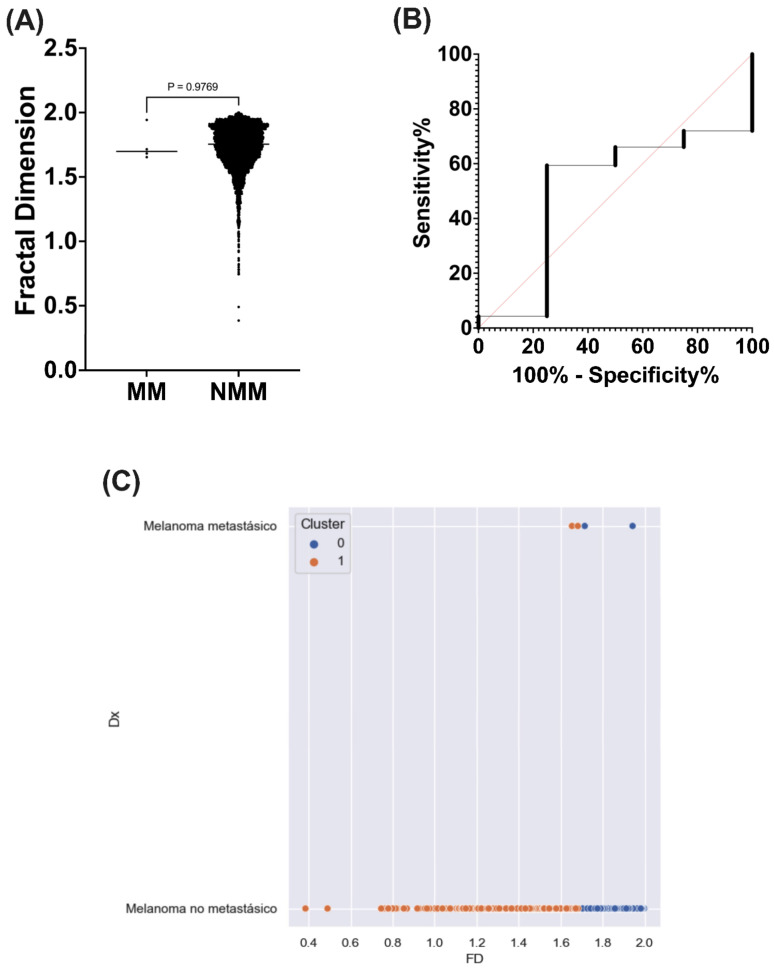
Fractal dimension analysis in metastatic or non-metastatic melanoma lesions. (**A**) Fractal dimension distribution values according to metastatic or non-metastatic melanoma lesions. (**B**) ROC analysis of fractal dimension for metastatic or non-metastatic melanoma lesion discrimination. (**C**) Dot plot showing the distribution of metastatic or non-metastatic melanoma lesions in the different clusters of the k-means analysis. MM = Metastatic melanoma lesions; NMM = non-metastatic melanoma lesion; PC = Principal component; Dx = Lesion classification into melanoma or non-melanoma lesions; FD = Fractal dimension value.

Subsequently, MM and NMM classification showed no significant differences (*p* = 0.976) (Figure 9A). 

#### Principal Component Analysis and k-Mean Analysis for Metastatic Melanoma or Non-Metastatic Melanoma Lesion Discrimination According to Fractal Dimension

PCA resulted in only one principal component with a loading value of −0.115 for MM and 0.292 for MNM. This may indicate an inversely proportional relationship to the fractal dimension values of this classification. To confirm this relationship, a Spearman correlation test was conducted, yielding a correlation coefficient of r = 0.012 (*p* = 0.346), thus dismissing the observations from the PCA analysis. Subsequently, ROC analysis of the fractal dimension to discriminate between MM and NMM lesions showed an area under the curve of 0.50 (95% CI = 0.23–0.76, *p* = 0.97), sensitivity of 59.39 and specificity of 75 (Figure 9B).

Additionally, a K-means test into two clusters yielding separation was observed between these lesions with a silhouette index of 0.58 and an inertia of 72.8. However, it is important to consider that the difficulty in classification by machine learning, and even the absence of significant differences between MM and NMM lesions, is limited by the small sample size included in MM lesions (Figure 9C).

## 4. Discussion

In the natural world, irregularity is a constant, with most structures in biological systems exhibiting irregular aggregations. The study of the aggregation and morphology of these structures is challenging and cannot be easily approached using classical Euclidean geometry. In cancer, cellular aggregation is evidently characterized by “quasi” random distributions, promoting an irregular and complex yet self-similar spatial arrangement. Therefore, the application of fractal geometry provides a suitable approach to understanding the morphological complexity of this group of diseases [28,29,30,31,32].

Obtaining imaging data for all types of neoplasms is currently challenging. Melanoma, from an imaging perspective, holds a privileged position, allowing for visual records without invasive approaches. This facilitates the study of the complexity of melanocytic lesion aggregation and promises a viable application for the diagnosis and screening of dermatological diseases [33]. Fractal dimension, by itself, proves useful in discriminating melanoma lesions from squamous cell carcinomas, nevi, vascular lesions and basal cell carcinomas. It is noteworthy that, although fractal dimension had been implemented in previous reports, they were limited to recognizing melanoma and non-melanoma lesions, reporting significant differences in this classification, consistent with the findings in this study.

This work proposes the study of the fractal dimension of dermatological lesions for classification using cutting-edge tools, such as unsupervised machine learning. While dermatoscopy enables more accurate diagnoses, its success remains operator-dependent, requiring precise training to identify lesions like melanoma. Therefore, the development of automated screening, classification and referral processes is crucial, given that skin cancers are the most common and deadly.

The use of AI for melanoma classification is not new, with research groups implementing technologies like convolutional neural networks (CNNs). However, these technologies have drawbacks, including high computational requirements and the need for high-quality images or videos for processing. Although they achieve classifications with around 90% accuracy using dermatoscopic images alone, other algorithms like K-nearest neighbor (KNN) exhibit lower classification accuracy than CNNs. Moreover, they require data extraction, such as color, roughness, three-dimensional arrangement, area and diameter of lesions, as well as features derived from the ABCDE criteria of melanoma. These AIs belong to supervised machine learning, relying on previously labeled data to define lesion groups, and may retain human bias in classification [34,35,36,37,38].

Given the above, this study focused on implementing unsupervised low-computational-requirements machine learning techniques to allow for free lesion classification by algorithms based on an objective and intrinsic characteristic of dermatoscopic images—their fractal dimension. With this strategy, specific high-quality clusters were identified by the K-means clustering technique, emphasizing the grouping of melanoma lesions, metastatic melanoma and Breslow 3 lesions without the need for prior labeling. This suggests that this technique can be implemented in clinical practice, with low acquisition-costs for lesion determination and may also align with histopathological classification criteria without the need for an invasive biopsy procedure. However, it is imperative to mention that this type of analysis does not aim to replace the gold standard of histopathology but intends to be an early screening tool applied at the primary care level for prompt referral to oncological dermatology services if needed.

It has been reported that the diagnosis of dermatological tumor lesions at the primary care level, by non-dermatologist physicians, currently exhibits low sensitivity (approximately 20%) and up to 94% specificity, alongside poor agreement. Coupled with extended referral periods and treatment initiation, malignant skin lesions have emerged as a significant health issue due to underdiagnosis [39]. Consequently, the results obtained in this study could enhance early diagnosis at the primary level by facilitating image processing of lesions through their fractal dimension.

**Figure 10:**
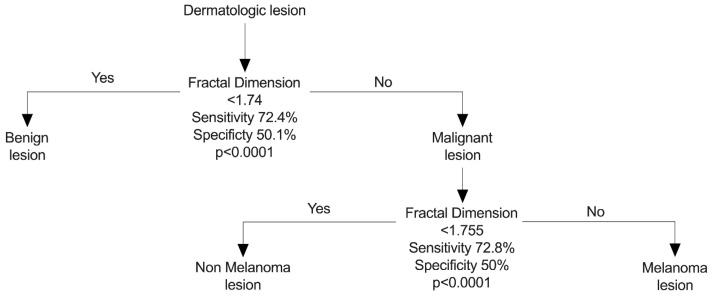
Suggested algorithm for dermatological lesion classification according to fractal dimension value.

Given the findings, two courses of action are proposed, both rooted in fractal dimension. The first, without the need for artificial intelligence classification methods, allows, based on ROC curve results, for differentiation between benign and malignant lesions (Sensitivity = 72.4% and Specificity = 50.1%). Within malignant lesions, it enables the distinction between melanoma and non-melanoma under a dichotomous classification method, with an approximate sensitivity of 72% and specificity of 50%. This approach can be applied at the primary care level by non-dermatologist physicians to enhance accurate skin tumor diagnosis. Unfortunately, due to the obtained statistical significance, this proposal can only extend to melanoma or non-melanoma lesions (Figure 10).

In this regard, the dichotomous method based on ROC curves, applied at the primary care level, potentially allows for the avoidance of underdiagnosis of melanoma lesions, increasing the current sensitivity from 20% to 72%; however, it decreases specificity from 94% to 50%. Nevertheless, even though this proposed method still encounters issues in identifying true negatives, it serves as a better model than the current one by identifying at least 72% of true positives. It is important to emphasize that this method is proposed for initial screening at the primary care level, and findings at this level should be validated by a dermatologist specialist.

The second proposal leverages artificial intelligence-based K-means classification capabilities, yielding clusters with a quality exceeding 0.80 in all cases. However, the application of this proposal is restricted to the development of mobile applications available for use by non-dermatologist physicians.

Although only the fractal dimension was implemented as a classification variable in this study, yielding robust clusters, it is suggested to enrich these methods with other clinical–pathological variables to enhance the quality of unsupervised artificial intelligence clusters.

## 5. Conclusions

The present study was able to determine that the fractal dimension, by itself, is an appropriate metric to discriminate between benign and malignant dermatological lesions, and between melanoma and non-melanoma lesions. However, this metric was not suitable to discriminate between metastatic and non-metastatic melanoma lesions.

On the other hand, it was also determined that the fractal dimension is sufficient to generate unsupervised artificial intelligence models that allow for a more efficient classification of dermatological lesions. However, it is suggested to strengthen the training of machine learning models with clinical–pathological parameters to evaluate whether it is possible to establish better classification algorithms based on even more efficient machine learning tools.

It is also important to consider that the development of this type of tool does not aim to replace the criteria of a dermatologist, but to provide an additional tool for the rapid and accurate diagnosis of skin diseases. This tool could be applied in remote locations, even through mobile applications, and therefore be easily accessible for use by medical personnel only.

## Data Availability

The data that support the findings of this study are available upon request from the corresponding author.

## References

[1] Yang Y., Xie F., Zhang H., Wang J., Liu J., Zhang Y., Ding H. (2023). Skin lesion classification based on two-modal images using a multi-scale fully-shared fusion network. Comput. Methods Programs Biomed..

[2] Wang Y., Su J., Xu Q., Zhong Y. (2023). A Collaborative Learning Model for Skin Lesion Segmentation and Classification. Diagnostics.

[3] Debelee T.G. (2023). Skin Lesion Classification and Detection Using Machine Learning Techniques: A Systematic Review. Diagnostics.

[4] Jenkins R.W., Fisher D.E. (2021). Treatment of Advanced Melanoma in 2020 and Beyond. J. Investig. Dermatol..

[5] Bobos M. (2021). Histopathologic classification and prognostic factors of melanoma: A 2021 update. Ital. J. Dermatol. Venereol..

[6] Pérez-Aldrete B.M., Matildes-Mariscal J.B., Gómez-Padilla F., Guevara-Gutiérrez E., Barrientos-García J.G., Hernández-Peralta S.L., Tlacuilo-Parra A. (2019). Cutaneous melanoma in patients from western Mexico: Clinical pathology characteristics and their relationship to prognosis. Australas. J. Dermatol..

[7] Lino-Silva L.S., Domínguez-Rodríguez J.A., Aguilar-Romero J.M., Martínez-Said H., Salcedo-Hernández R.A., García-Pérez L., Herrera-Gómez Á., Cuellar-Hubbe M. (2016). Melanoma in Mexico: Clinicopathologic Features in a Population with Predominance of Acral Lentiginous Subtype. Ann. Surg. Oncol..

[8] Breslow A. (1975). Tumor thickness, level of invasion and node dissection in stage I cutaneous melanoma. Ann. Surg..

[9] Puckett Y., Wilson A.M., Farci F., Thevenin C. (2017). Melanoma Pathology.

[10] Heistein J.B., Acharya U. (2022). Malignant Melanoma.

[11] Carbonetto S.H., Lew S.E. Characterization of border structure using fractal dimension in melanomas. Proceedings of the 2010 Annual International Conference of the IEEE Engineering in Medicine and Biology.

[12] Mehmood A., Gulzar Y., Ilyas Q.M., Jabbari A., Ahmad M., Iqbal S. (2023). SBXception: A Shallower and Broader Xception Architecture for Efficient Classification of Skin Lesions. Cancers.

[13] Kumar A.B., Peters M.S., Jakub J.W., Harmsen W., Lee Baum C. (2022). Primary cutaneous melanoma features predict development of intransit metastases/satellite lesions: Mayo Clinic experience 2010–2014. J. Am. Acad. Dermatol..

[14] Dinnes J., Deeks J.J., Chuchu N., Ferrante di Ruffano L., Matin R.N., Thomson D.R., Wong K.Y., Aldridge R.B., Abbott R., Fawzy M. (2018). Dermoscopy, with and without visual inspection, for diagnosing melanoma in adults. Cochrane Database Syst. Rev..

[15] Holmes G.A., Vassantachart J.M., Limone B.A., Zumwalt M., Hirokane J., Jacob S.E. (2018). Using Dermoscopy to Identify Melanoma and Improve Diagnostic Discrimination. Fed. Pract..

[16] Huang S., Yang J., Fong S., Zhao Q. (2020). Artificial intelligence in cancer diagnosis and prognosis: Opportunities and challenges. Cancer Lett..

[17] Cuocolo R., Caruso M., Perillo T., Ugga L., Petretta M. (2020). Machine Learning in oncology: A clinical appraisal. Cancer Lett..

[18] Thomsen K., Iversen L., Titlestad T.L., Winther O. (2020). Systematic review of machine learning for diagnosis and prognosis in dermatology. J. Dermatol. Treat..

[19] Breki C.M., Dimitrakopoulou-Strauss A., Hassel J., Theoharis T., Sachpekidis C., Pan L., Provata A. (2016). Fractal and multifractal analysis of PET/CT images of metastatic melanoma before and after treatment with ipilimumab. EJNMMI Res..

[20] Maier T., Kulichova D., Schotten K., Astrid R., Ruzicka T., Berking C., Udrea A. (2015). Accuracy of a smartphone application using fractal image analysis of pigmented moles compared to clinical diagnosis and histological result. J. Eur. Acad. Dermatol. Venereol..

[21] Gulzar Y., Khan S.A. (2022). Skin Lesion Segmentation Based on Vision Transformers and Convolutional Neural Networks—A Comparative Study. Appl. Sci..

[22] Song Z., Luo W., Shi Q. (2022). Res-CDD-Net: A Network with Multi-Scale Attention and Optimized Decoding Path for Skin Lesion Segmentation. Electronics.

[23] Khan S.A., Gulzar Y., Turaev S., Peng Y.S. (2021). A Modified HSIFT Descriptor for Medical Image Classification of Anatomy Objects. Symmetry.

[24] Hasan M.d.K., Ahamad M.d.A., Yap C.H., Yang G. (2023). A survey, review, and future trends of skin lesion segmentation and classification. Comput. Biol. Med..

[25] Ren Z., Wang S., Zhang Y. (2023). Weakly supervised machine learning. CAAI Trans. Intell. Technol..

[26] Nath Mohalder R., Hossain M.A., Hossain N. (2024). Classifying the supervised machine learning and comparing the performances of the algorithms. Int. J. Adv. Res. (Indore).

[27] Esther Varma C., Prasad P.S. (2023). Supervised and Unsupervised Machine Learning Approaches—A Survey. ICDSMLA 2021.

[28] Tsonis A.A., Biham O., Malcai O., Lidar D.A., Avnir D. (1998). Fractality in Nature. Science.

[29] Legaria-Peña J.U., Sánchez-Morales F., Cortés-Poza Y. (2023). Evaluation of entropy and fractal dimension as biomarkers for tumor growth and treatment response using cellular automata. J. Theor. Biol..

[30] Losa G.A. (2009). The fractal geometry of life. Riv. Biol..

[31] Heymans O., Fissette J., Vico P., Blacher S., Masset D., Brouers F. (2000). Is fractal geometry useful in medicine and biomedical sciences?. Med. Hypotheses.

[32] Losa G.A. (2002). Fractal morphometry of cell complexity. Riv. Biol..

[33] Bizzarri M., Giuliani A., Cucina A., D’Anselmi F., Soto A.M., Sonnenschein C. (2011). Fractal analysis in a systems biology approach to cancer. Semin. Cancer Biol..

[34] Chaugule B., Bomble K., Jundare S., Maske N., Gagare V. (2023). Skin Melanoma Cancer Detection and Classification using Machine Learning. Int. J. Sci. Res. Sci. Technol..

[35] Moldovanu S., Damian Michis F.A., Biswas K.C., Culea-Florescu A., Moraru L. (2021). Skin Lesion Classification Based on Surface Fractal Dimensions and Statistical Color Cluster Features Using an Ensemble of Machine Learning Techniques. Cancers.

[36] Patil S., Moafa I.H., Mosa Alfaifi M., Abdu A.M., Jafer M.A., Raju K.L., Raj A.T., Sait S.M. (2020). Reviewing the Role of Artificial Intelligence in Cancer. Asian Pac. J. Cancer Biol..

[37] Bhinder B., Gilvary C., Madhukar N.S., Elemento O. (2021). Artificial Intelligence in Cancer Research and Precision Medicine. Cancer Discov..

[38] Coccia M. (2020). Deep learning technology for improving cancer care in society: New directions in cancer imaging driven by artificial intelligence. Technol. Soc..

[39] Castillo-Arenas E., Garrido V., Serrano-Ortega S. (2014). Motivos dermatológicos de consulta en atención primaria. Análisis Demanda Deriv. Actas Dermosifiliogr..

